# Development of Cancer in Patients With Heart Failure: How Systemic Inflammation Can Lay the Groundwork

**DOI:** 10.3389/fcvm.2020.598384

**Published:** 2020-10-26

**Authors:** Simonetta Ausoni, Giuseppe Azzarello

**Affiliations:** ^1^Department of Biomedical Sciences, University of Padua, Padua, Italy; ^2^Local Health Unit 3 Serenissima, Department of Medical Oncology, Mirano Hospital, Venice, Italy

**Keywords:** cancer, heart failure, systemic inflammation, cardiokines, carcinogenesis

## Abstract

In the last decade, cardiologists and oncologists have provided clinical and experimental evidence that cancer, and not only chemotherapeutic agents, can cause detrimental effects on heart structure and function, a consequence that has serious clinical implications for patient management. In parallel, the intriguing idea that heart failure (HF) may be an oncogenic condition has also received growing attention. A number of epidemiological and clinical studies have reported that patients with HF have a higher risk of developing cancer. Chronic low-grade systemic inflammation has been proposed as a major pathophysiological process linking the failing heart to the multi-step process of carcinogenesis. According to this view, pro-inflammatory mediators secreted by the damaged heart generate a favorable milieu that promotes tumor development and accelerates malignant transformation. HF-associated inflammation synergizes with tumor-associated inflammation, so that over time it is no longer possible to distinguish the effects of one or the other. Experimental studies have just begun to search for the molecular effectors of this process, with the ultimate goal that of identifying mechanisms suitable for anti-cancer target therapy to reduce the risk of incident cancer in patients already affected by HF. In this review we critically discuss strengths and limitations of clinical and experimental studies that support a causal relationship between HF and cancer, and focus on HF-associated inflammation, cardiokines and their endocrine functions linking one and the other disease.

## Introduction

Cancer and cardiovascular diseases are the leading causes of death worldwide, raising the question of whether these diseases have intersecting pathogenetic mechanisms. Clinical and experimental studies have demonstrated that cancer patients develop cardiac disease not only as a consequence of cardiotoxicity induced by chemotherapies, but also because tumor cells release soluble factors that affect various organs at a distance, including the heart (1, 2). Cancer-derived pro-inflammatory molecules cause cardiomyocyte atrophy and tissue remodeling, which can degenerate to cachexia and HF (3–5).

Over the past 10 years, attention has also been moving in an opposite direction, intriguingly posing the question as to whether injured myocardium might of itself trigger cancer development. Thus, cardio-oncology, the discipline that traditionally used to investigate radio and chemotherapy-induced cardiotoxicity, has crossed the orthodox boundaries with expansion of interest in the possibility of bidirectional cancer-heart communication (2, 6–10).

The causal relationship between HF and cancer has been a matter of investigation, and also controversial results, in epidemiologic and clinical studies. An elevated risk of incident cancer has been reported in some studies (11–14), raising the hypothesis that HF may predispose to cancer and that a common milieu characterized by a low-grade chronic inflammation could mediate both conditions. In search of proof of the principle of HF causing cancer, an experimental model of HF and cancer has been also developed (15).

In this review, we provide a critical reappraisal of clinical and experimental studies linking HF and cancer moving from an “oncological point of view” rather than from a “cardiological point of view,” where the latter has been the case in most previous reviews. We discuss the complex HF-associated inflammatory phenotype, the role of secreted cardiokines in generating a systemic inflammatory condition and the potential role and timing of HF-associated inflammation in the multistep process of carcinogenesis. Finally, we identify challenges for future investigation that will be aimed at unraveling possible causal effectors connecting HF and incident cancer, and possibly offer target therapies to reduce the risk of cancer in HF patients.

## Cancer Development in HF Patients: Points and Counterpoints in Clinical Studies

HF is a life-threatening clinical syndrome characterized by “inherited or acquired abnormality of cardiac structure and function, associated with a constellation of clinical symptoms (dyspnea and fatigue) and signs (edema and rales)” that often severely compromise the quality and duration of life (16). Guidelines from the North American and European cardiology societies classify HF based on the proportion of blood pumped from the ventricles, i.e., the ejection fraction (EF). Accordingly, they distinguish HF with reduced EF (HFrEF) and HF with preserved EF (HFpEF). HFrEF is mainly caused by coronary artery disease followed by myocardial infarction and loss of cardiomyocytes. In this disease the ventricular myocardium can no longer contract properly and EF is severely reduced. Conversely, HFpEF is promoted by multiple comorbidities, such as obesity, diabetes, atherosclerosis, and arterial hypertension. In HFpEF ventricular contractility is preserved, but the ventricular myocardium fails to relax during diastole and consequently filling pressure is increased (17).

In 2013, Hasin et al. (11) published a retrospective analysis with the first strong epidemiological evidence that patients with HFpEF and HFrEF had a higher likelihood of diagnosis of/or of dying from cancer compared with community controls. The study showed a 70% increase in cancer risk over a prolonged median follow-up of more than 5 years, a value that was maintained after correction for comorbidities and risk factors. In a second study on 5,000 patients with chronic HF cancer incidence was 4-fold higher than in control patients. Values positively correlated with circulating levels of brain natriuretic peptide (BNP), a marker of cardiac stress response and tissue damage (18). A large community study based on the Danish National Registries also reported that patients with HF, mainly as HFpEF, had a greater risk of incident cancer (significant incidence rate ratio of 1.24 for all major types of cancer except for prostate cancer) and a worse prognosis (13). While limited by its observational nature, leaving potential only for the generation of hypotheses, this study's strengths were the number of patients examined and the appropriate follow-up. In a prospective cohort-study, Hasin et al. (14) supported previous findings showing that patients who developed HF after myocardial infarction had an increased risk of incident cancer, with a hazard ratio for the association of 2.16, adjusted for age, sex, and comorbidities. An association between HF and incident cancer risk has been also confirmed in a recent pooled analysis (19).

Other clinical studies have questioned the causal relationship between HF and incident cancer providing opposite evidences. In a first *post-hoc* analysis (Physicians' Health Studies I and II) conducted on a large male population included in two controlled trials on the preventive effect of aspirin and vitamin supplement on incidence of cancer and HF, no correlation was found between the two diseases (20). Though supported by adequate sample size, follow-up and statistics, there were elements of weakness in the study concerned, such as HF diagnosis based on self-reported questionnaires rather than on established criteria, as well as its restriction to a male population. Two additional studies reported no causal relationship between HF and increased cancer risk. In the former study conducted on a large cohort of patients previously affected by myocardial infarction, the authors concluded that occult cancer and shared risk factors were likely responsible for the higher cancer incidence (21). In the second study, the authors attributed the slightly increased cancer risk to baseline comorbidities. Finally, a recent review and meta-analysis focusing on the association between myocardial infarction and cancer reported a 9.5% estimated cancer incidence after myocardial infarction, a value that attained statistical significance only in female patients, and that was substantially restricted to breast cancer (22).

On the basis of the aforementioned results it is clear that the impact of HF on cancer incidence is a complicated issue still open to investigation (23), hopefully through prospective clinical trials with large samples and long-term follow-up. The following biases need to be carefully considered before designing new clinical studies.

1. COMMON RISK FACTORS. The association between HF and cancer could depend on common risk factors, since many patients enrolled in the clinical studies had several comorbidities, such as obesity, diabetes, or hypertension. In addition, smoking and alcohol consumption may have contributed to a confounding of the results. Even though previous epidemiological studies suggest that the association is maintained after data correction for the main risk factors, this key point needs to be duly weighed in well-designed prospective studies. These studies could, at the same time, clarify whether cancer risk in HF patients is generalized or is restricted to specific cancer subtypes.
2. DETECTION BIAS. The association between HF and cancer may be due to the so-called detection bias, i.e., the closest clinical-instrumental follow-up applied to HF patients. A preliminary answer to this question emerges from studies showing that the highest incidence of cancer was recorded well-beyond the first year after diagnosis of HF, which is generally the period of greatest intensity of cardiology controls (11, 14, 20).
3. CARDIOLOGIC THERAPIES. The potential carcinogenic effect of some cardiologic therapies for HF has also been called into question. Treatment of hypertensive patients with Telmisartan, an angiotensin-receptor blocking drug, was found to be associated with a modestly raised risk of new cancer, and in particular lung carcinomas (24–26). Regarding statins, the inhibitors of 3-hydroxy-3-methyl-glutaryl-coenzyme A (HMG-CoA) and cholesterol synthesis, their carcinogenic risk remains controversial (27). Initial studies in the late 1990s reported an increased cancer-related mortality in patients treated with statins (28). However, further studies did not confirm these data (29), indeed preclinical research demonstrated that statins inhibit tumor growth, induce apoptosis in some cancer types and have a general anti-inflammatory and anti-oxidant protective effect (30).
4. DIAGNOSTIC PROCEDURES of HF and CANCER RISK. HF patients undergo several diagnostic procedures with exposure to ionizing radiations and potential risk of cancer. For instance, the risk associated with coronary angiography is not negligible and justifies adoption of alternative diagnostic procedures (31). Electrocardiogram (ECG)-gated computed tomography angiography (CTA), in the form of prospective analysis, represents an alternative option for radiation safety. Prospective gating only acquires images during the cardiac diastole, instead of throughout the entire cardiac cycle like retrospective gating. This procedure allows high image quality and reduction of the effective radiation dose by up to 90%, thus lowering the cancer induction risk in sensitive organs to 0.13% (32).

## Systemic Inflammation Can Link HF and Incident Cancer

### A Complex Cardio-Inflammatory Phenotype in HF

**Figure 1:**
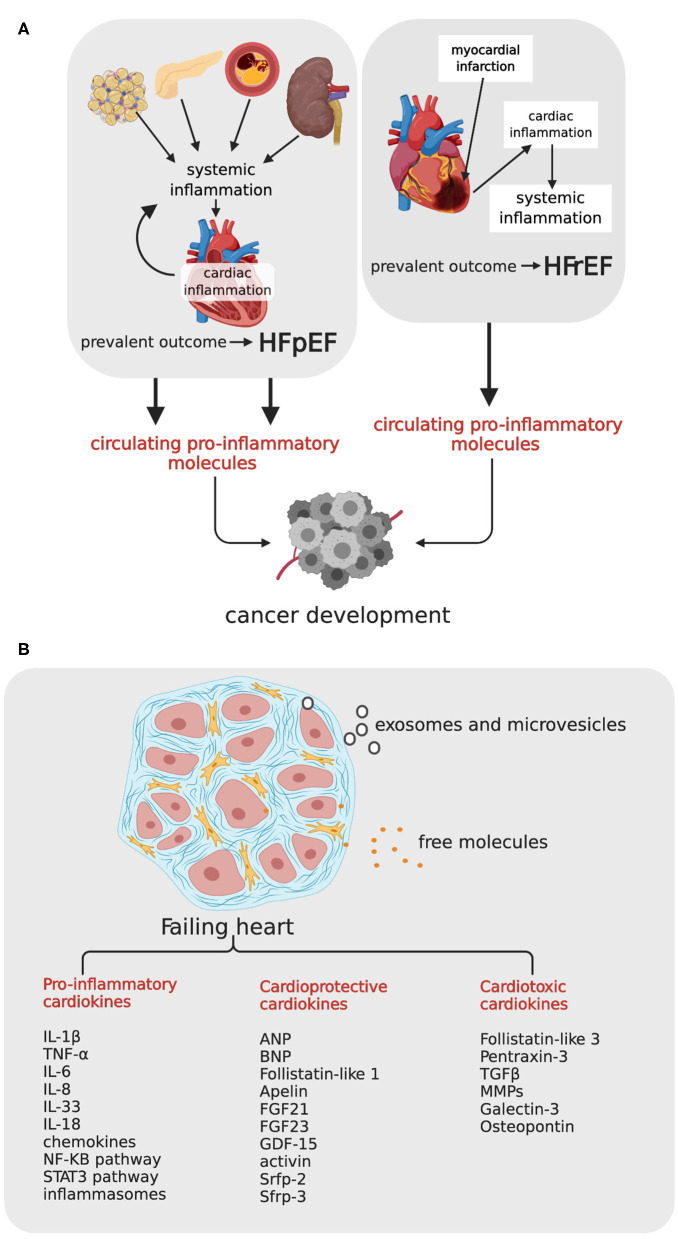
Heart failure, cardiokines, and relationship with incident cancer. **(A)** HFrEF and HFpEF can potentially promote carcinogenesis by means of circulating pro-inflammatory molecules. In HFrEF consequent to myocardial infarction (right side), heart damage activates cardiac inflammation, which occasionally induces a chronic systemic inflammation and a persistent tissue damage; in HFpEF (left side), comorbidities like obesity, diabetes, atherosclerosis and hypertension, induce a systemic inflammatory state, with consequent cardiac inflammation, and functional impairment. HFpEF, Hear Failure with Preserved Ejection Fraction; HFrEF, Heart Failure with reduced Ejection Fraction. **(B)** The failing heart, schematically represented as a myocardial tissue section with cardiomyocytes surrounded by fibrotic matrix, secretes a variety of cardiokines, either as free molecules or as small microvesicles and exosomes. TNF-α, tumor necrosis factor-α; IL-1β, interleuchin-1β; IL-6, interleuchin-6; IL-33, interleuchin-33; IL-17, interleuchin-17; MMPs, matrix metalloproteases; TGF-β, transforming growth factor-β; ANP, Atrial Natriuretic Peptide; BNP, Brain Natriuretic Peptide; FGF21, fibroblasts growth factor 21, FGF23, fibroblasts growth factor 23; GDF-15, TGF-β related family members growth differentiation factor-15, Srfp-2 and Srfp-3, secreted frizzled related proteins 2 and 3. Created with Biorender.com.

Extensive evidence has shown that HF is associated with a chronic inflammatory state and activation of immune response. Inflammation arises locally as a consequence of myocardial injury in HFrEF, and systemically as a consequence of various comorbidities in HFpEF (33, 34) (Figure 1A). In HFrEF, dying or stressed cells release damage-associated molecular patterns (DAMPS), which elicit a robust inflammatory and immune response. Inflammatory cells and also cardiomyocytes, endothelial cells, and cardiac fibroblasts start producing a variety of cytokines—including IL-1β, IL-6, TNF-α, IL-8, IL-18, IL-33—chemokines, and proteins of the pro-inflammatory signaling pathways. Inflammatory mediators blunt responsiveness of cardiomyocytes at multiple levels, decreasing sarcoplasmic reticulum calcium load, and depressing contractility, reducing ATP synthesis, increasing production of oxygen (ROS) and nitrogen (RNS) reactive species (35) and reorienting metabolism from oxidative to glycolytic (so-called metabolic remodeling) (36). Over the weeks, cardiac inflammation exits in repair and tissue remodeling, but inflammation may inexplicably become chronic in some patients, and exacerbate myocardial damage. Given the detrimental effects of an excessive and persistent pro-inflammatory response in HFrEF, therapeutic strategies for attenuating the initial pro-inflammatory response are underway.

In HFpEF, inflammation drives the development of HF through another sequence of events (37). Comorbidities induce a systemic pro-inflammatory state. Myocardial disease begins with coronary endothelial dysfunction and expression of adhesion molecules VCAMs and selectins, and attraction of infiltrating leukocytes. Endothelial cells start producing ROS, and ROS trigger a negative cascade of events with downregulation of nitric oxide (NO) production, decreased NO-stimulated cyclic guanosine monophosphate (cGMP) levels, and decreased protein kinase G (PKG) activity. This sequence culminates in PKG-dependent hypophosphorylation of titin, the protein that regulates cardiomyocyte stiffness, depending on its phosphorylation state. Stiff cardiomyocytes, in association with interstitial fibrosis and capillary rarefaction, contribute to impaired diastolic relaxation, a hallmark of HFpEF (38). Thus, oxidative stress and inflammation, whether the consequence or the cause of HF, generate a chronic systemic condition with adverse clinical outcome. Although low-grade chronic systemic inflammation is often clinically silent, its consequences may also increase the risk of different cancers. In line with these observation, genetic polymorphisms in genes encoding IL-1β, IL-6, IL-8, and anti-inflammatory IL-10, have been shown to predispose affected individuals to cancer (39).

### A Cardiac Secretome With Carcinogenic Potential

Like other organs, the heart secretes a variety of molecules in response to metabolic and hemodynamic stress. The “cardiac secretome” includes peptides, proteins and coding, and non-coding RNAs released either as free molecules or as microvesicles and exosomes. The expression profile of secreted peptides and proteins—called cardiokines—changes considerably under pathological conditions (40–43). Cardiokines produced by the failing heart include primary citokines and chemokines of inflammation (mostly with detrimental effects for cardiomyocytes), and also proteins involved in stress response, regulation of apoptosis, tissue repair, cardiac remodeling, and angiogenesis (Figure 1B). A large number of cardiokines are functionally intended to maintain cardiac homeostasis and are therefore cardioprotective. This group includes, among others, atrial natriuretic peptide (ANP) and BNP (18), fibroblasts growth factor 21 (FGF21) (44) and 23 (FGF23) (45), TGF-β related family members growth differentiation factor (GDF)-15 (46, 47), activin (48), secreted frizzled related proteins 2 (Srfp-2) (49, 50) and 3 (Srfp-3) (51, 52), and follistatin-like protein-1 (53). Other cardiokines, such as pro-inflammatory follistatin-like protein-3 (54), pentraxin 3 (55), TGF-β matrix metalloproteases (MMPs) (56), as well as other proteins involved in extracellular matrix degradation and remodeling, are cardiotoxic. Up-regulation of these proteins in HF patients is predictive of an adverse outcome.

Since the discovery of natriuretic peptides, it has become evident that cardiokines have a great influence in altering the homeostasis of distant tissues. To test the hypothesis that cardiokines released by the failing heart could induce carcinogenesis Meijers et al. induced myocardial infarction in APC^min^ mice, a murine model genetically predisposed to develop intestinal polyposis (15). Mice developed more than twice the tumor load as compared to sham-operated controls. To rule out hemodynamic impairment as a causative mechanism for increased tumors, the investigators heterotopically transplanted an additional heart, with previously induced acute myocardial infarction, in the same APC^min^ mouse strain. Tumor load was increased in the presence of an infarcted transplanted heart, but not with an intact heart, indicating that the pro-carcinogenic effect is due to the failing heart. Using *in silico* analysis, Meijers et al. identified five potential circulating factors responsible for polyposis, namely Fibronectin, SerpinA3, SerpinA1, Ceruloplasmin, and Paraonase1. The same factors were also up-regulated in HF patients enrolled in the PREVEND study (Prevention of Renal and Vascular End-Stage Disease), together with inflammatory molecules (C-reactive protein, pro-endothelin, pro-calcitonin, and pro-adrenomedullin) and cardioprotective ANP and BNP. Of the five markers identified in APC^min^mice, only SerpinA3 and SerpinA1 were able to stimulate proliferation of colon cancer cell line HT-29 *in vitro* and no test *in vivo* was performed to confirm the results. Thus, Maijers study did not provide conclusive evidence that the identified cardiokines were the true effectors of tumorigenesis.

Other cardiokines with high expression in HF patients have shown tumorigenic potential in previous studies, and are therefore candidates for further investigation. Apelin, a ligand of the G protein-coupled receptor APJ (57), which reduces oxidative stress and prevents cardiac hypertrophy in HF (58), stimulates proliferation of cancer cells in cholangiocarcinoma (CAA) (59), non-small cell lung cancer (NSCLC) (60), gastric cancer (61), prostate cancer (62), ovarian cancer (63), and oral squamous cell carcinoma (64). Similarly, Galectin-3, a carbohydrate-binding protein with pro-inflammatory and pro-fibrotic function in the heart (65), stimulates tumor progression and metastatization (66), and osteopontin, a glycoprotein highly expressed in the post-ischemic heart (67) stimulates primary tumor cell proliferation, angiogenesis, and epithelial- mesenchymal transition (EMT) (68).

### How Cardiokines Elicit Carcinogenesis: A Critical Reappraisal

More than 100 years ago, Virchow emphasized the association between chronic inflammation and the onset of cancer, starting from the histopathological observation that tumors arise from sites with dense “lymphoreticular infiltration” (69). Association of infection, inflammation and cancer has been reported for a variety of clinical conditions, including inflammatory bowel disease for colorectal cancer, Hepatitis B and C for hepatocarcinoma, schistosoma–induced bladder and colorectal cancer, and *Helicobacter pylori* infection for gastric mucosa-associated lymphoid tissue lymphoma. In addition, environmental and lifestyle factors that predispose to local inflammation, such as inhaled fine particles, asbestos, tobacco smoke, and alcohol consumption, have been shown to induce cancer (70).

The concept of inflammation as a hallmark of cancer (71, 72) has been proposed for local inflammation and more recently also for systemic inflammation associated with a variety of chronic diseases (73, 74). For instance, metabolic syndrome, a cluster of disorders including obesity, hypertension, dyslipidemia and insulin resistance, predisposes to cancer development, mainly through activation of a chronic inflammatory state sustained by adipose cell-released cytokines (adipokines). Adipokines and locally generated ROS prime both premalignant cells and the microenvironment to facilitate oncogenic transformation (75, 76). In addition to acting locally, adipokines, and the chronic inflammatory microenvironment also mediate crosstalk with distant tissues, thus reflecting the epidemiological evidence that obesity is significantly associated with specific tumor subtypes, mainly breast and pancreas cancer (77).

The hypothesis that a low-grade chronic inflammation has carcinogenic potential has raised the question of whether anti-inflammatory therapies may reduce the risk of cancer. In the CANTOS trial (Canakinumab Anti-inflammatory Thrombosis Outcome Study), administration of IL-1β-targeting antibody Canakinumab reduced HF-related hospitalization and mortality in patients with previous myocardial infarction (78). In a retrospective analysis, the same authors found a marked reduction in the hazard ratio of lung cancer incidence in patients treated with 150 mg and 300 mg of Canakinumab, compared with placebo controls (79). This finding opened a new research path aimed at investigating the therapeutic potential of anti-inflammatory drugs in cancer patients. Conventional non-steroid anti-inflammatory drugs (NSAIDs), including aspirin, have been also tested. Numerous studies observed that people who regularly take low doses of aspirin may have reduced risks of being diagnosed with or dying from cancer (80). Controlled clinical trials with patients treated with aspirin vs. placebo suggested a long-term risk reduction in the onset of any neoplasm, particularly of gastric, esophageal, colorectal, pancreatic, ovarian, endometrial, breast and prostate cancer, and small intestine neuroendocrine tumors (81, 82). However, the very recent ASPREE study demonstrated that in older adults aspirin had an adverse effect on later stages of cancer evolution, with a higher incidence of metastatic cancers and a higher risk of death (83). These data indicate that it is necessary to remain cautious in defining the preventive anti-cancer effect of aspirin for wide application.

**Figure 2:**
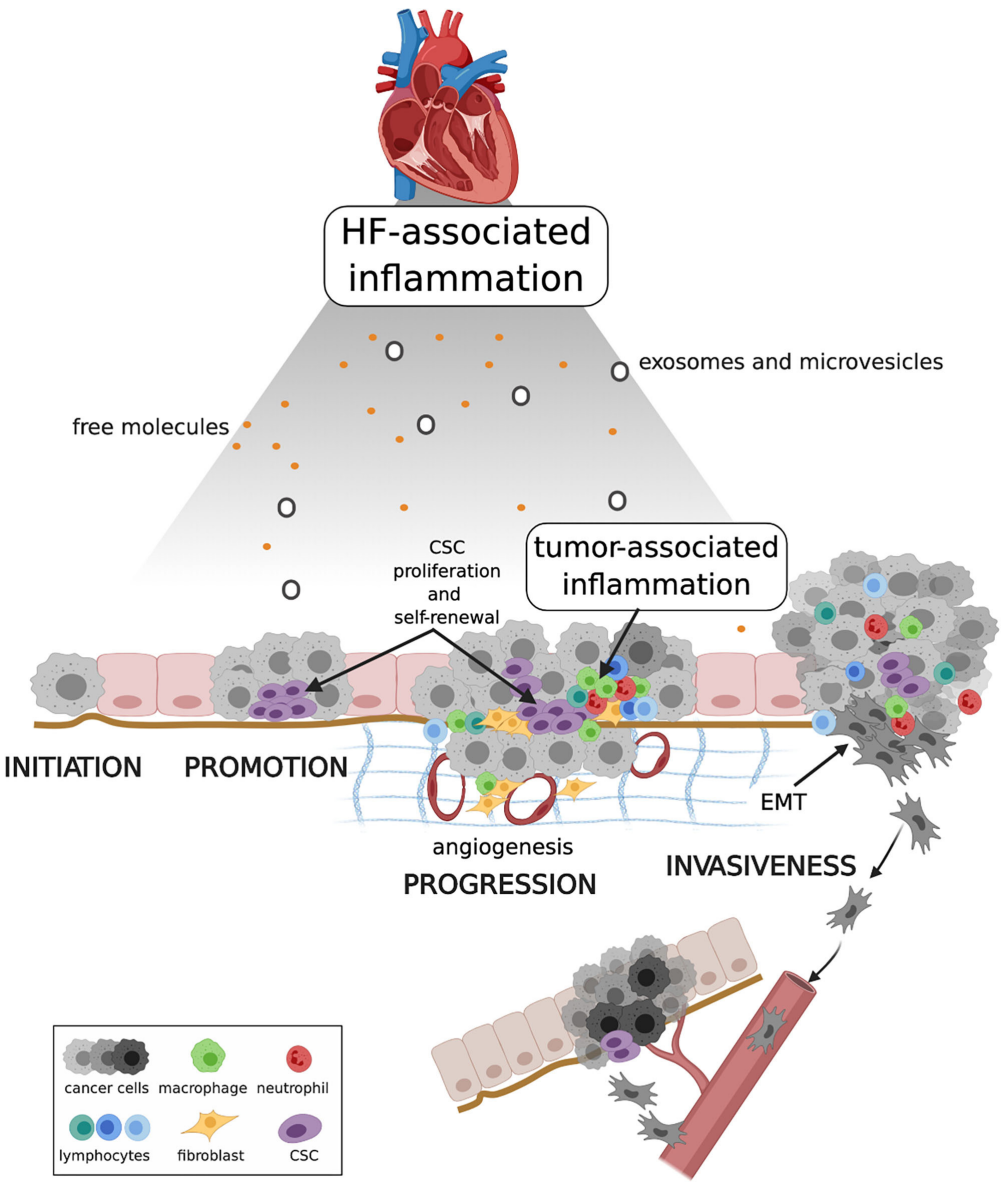
Proposed mechanism linking HF, systemic inflammation and carcinogenesis. HF-associated inflammation can have a direct effect on cancer initiation, and on pre-malignant cell proliferation and resistance to death during cancer promotion. HF-associated inflammation, together with tumor-associated inflammation, shapes the tumor microenvironment, enhancing CSC proliferation and self-renewal, and stimulating angiogenesis and EMT. Collectively these events contribute to tumor progression and invasiveness. CSC, cancer stem cell; EMT, epithelial-mesenchymal transition. Created with Biorender.com.

Although results obtained in the CANTOS study and in the experimental model described by Meijers et al. (15) will require further validation, they undoubtedly have the merit of raising the crucial question of whether cardiokines released by the failing heart promote carcinogenesis and if they do what are the mechanisms. The experimental APC^min^ mouse model is already predisposed to polyposis because it carries a truncating mutation at codon 850 of the *Adenomatous polyposis coli* (Apc) gene. Mice with heterozygous mutation develop ~30 small intestinal polyps that progressively cause colon obstruction and death in mice within 3 months. Occasionally, polyps progress to adenocarcinoma, and never to metastatic disease (84). One might therefore argue that myocardial infarction and the consequent inflammatory state contribute to speed up a process that would take place in any case. This apparent limitation could nonetheless shed light on what is the most plausible pathogenetic mechanism linking HF and incident cancer (Figure 2). In a low-grade chronic inflammatory state, a large number of molecular pro-inflammatory mediators and inflammatory cells circulate in the bloodstream. Inflammation produce ROS and RNS, which are powerful mutagens and can cause DNA double strand breaks and genomic instability (85, 86). When tumor initiation is nor preceded by systemic inflammation, as it is in APC^min^ mice, systemic inflammation can nonetheless drive cell proliferation and accelerate carcinogenesis (87). Once generated, the tumor bulk recruits its own inflammatory milieu, which is in continuous cross-talk with HF-associated inflammation, so that over time it is no longer possible to distinguish the effects of one or the other. Inflammation contributes to tumor promotion by editing the tumor microenvironment. Low levels of IL-1β, TNF-α, and IL-6 stimulate angiogenesis, and IL-6 modulates polarization of tumor-associated macrophages (TAM) from M1 (a tumor-eliminating phenotype) to M2 (an anti-inflammatory and tumor-promoting phenotype) (88, 89). Inflammation also regulates cancer stem cells (CSCs) cell cycle and epithelial-mesenchymal transition (EMT), two major events in tumor progression. CSCs, namely those rare immortal cells of the tumor bulk with the capacity for self-renewal, multipotency, tumorigenicity, and quiescence (90), proliferate in response to activation of the NF-kB and STAT3 pathways (91), and EMT, a prerequisite for invasiveness, is favored by exposure of tumor cells to TNF-α, IL-6, and TGF-β (92, 93). Systemic inflammation also reactivates quiescent cancer cells. Experimental studies have proved that sustained lung inflammation and the accompanying formation of neutrophil extracellular traps (NETs) convert dormant cancer cells to aggressive lung metastases through NET-mediated remodeling of the extracellular matrix (94). Finally, intravasation and extravasation of cancer cells during metastatic spread is favored by endothelial dysfunction and expression of adhesion molecules for leucocytes, a process sustained by pro-inflammatory cytokines. HF-associated inflammation thus can play an instrumental role in the control of all steps of carcinogenesis and support the causal relationship between HF and cancer.

## Conclusion and Open Issues

A number of epidemiological and clinical studies have reported that patients with HF have a higher risk of developing cancer and a chronic low-grade systemic inflammation has been proposed as the major pathophysiological process linking HF and carcinogenesis. The relationship between HF, systemic inflammation, and cancer is complicated and a unifying picture is still lacking (23). In particular, there are outstanding questions that cardiologists and oncologists have yet to answer through cooperative action. Are all HF variants, including the majority of HFpEF, prone to develop incident cancer? Most clinical studies describe HF consequent to myocardial infarction, but the contribution of other HF variants remains largely unexplored. Another key question concerns HF-associated inflammation. Do we deal with an organ-specific and disease-specific inflammation or is it on a par with any other inflammation in its carcinogenic potential? Which cardiokines secreted by the failing heart truly promote carcinogenesis and are therefore worse prognostic? To address these issues a more detailed characterization of the inflammatory profile of cardiokines in various HFrEF and HFpEF is eagerly awaited. In addition, mouse models establishing the carcinogenic potential of specific cardiokines *in vivo* are essential. A large number of transgenic mice with genetically-induced cardiomyopathies have been characterized, some of which develop progressive HF in a reasonably large window of time that permits cancer development, either genetically-induced or consequent to tumor cell injection (95). These models should be considered when designing future experiments.

For the perspective of preventing cancer in HF patients, cardio-oncologists should consider the possibility of identifying a targetable “cardio-inflammatory” phenotype in HF patients. This approach has important clinical implications in terms of patient stratification, risk assessment, and the determination of specific therapeutic algorithms. So far, therapeutic agents used to manipulate inflammation in patients with HF and cancer have achieved modest results, possibly because these agents may be more effective in preventing cancer or in treating initial cancer, rather than advanced cancer. Targeting IL-1β with Canakinumab opened a new era in the use of selective anti-inflammatory therapies in cardiovascular diseases, but its potential in cancer prevention needs to be further explored. Anti-inflammatory therapies affect immunosuppression and modulation of immunosuppression is one of the most promising therapeutic strategies for cancer (96). Thus, the use of anti-inflammatory or anti-immune agents, alone or in combination, represent a logical focus of therapeutic intervention to establish whether combating inflammation truly reduces the risk of cancer in HF patients.

## Author Contributions

SA contributed to the conceptualization, writing and editing of the manuscript, and to illustration design. GA contributed to the conceptualization and writing of the manuscript. All authors approved the submitted version.

## Conflict of Interest

The authors declare that the research was conducted in the absence of any commercial or financial relationships that could be construed as a potential conflict of interest.
